# Regional Variation in Deescalated Therapy in Older Adults With Early-Stage Breast Cancer

**DOI:** 10.1001/jamanetworkopen.2024.41152

**Published:** 2024-10-24

**Authors:** Christina A. Minami, Tanujit Dey, Yu-Jen Chen, Rachel A. Freedman, Eliza H. Lorentzen, Tari A. King, Elizabeth A. Mittendorf, Mara A. Schonberg

**Affiliations:** 1 Division of Breast Surgery, Department of Surgery, Brigham and Women’s Hospital, Boston, Massachusetts; 2 Breast Oncology Program, Dana-Farber Brigham Cancer Center, Boston, Massachusetts; 3 Harvard Medical School, Boston, Massachusetts; 4 Center for Surgery and Public Health, Brigham and Women’s Hospital, Boston, Massachusetts; 5 Medical Oncology, Dana-Farber Cancer Institute, Boston, Massachusetts; 6 Department of Medicine, Beth Israel Deaconess Medical Center, Boston, Massachusetts

## Abstract

**Importance:**

Although trial data support the omission of axillary surgery and radiation therapy (RT) in women aged 70 years or older with T1N0 hormone receptor–positive (HR^+^) breast cancer, potential overtreatment in older adults with frailty persists.

**Objective:**

To determine how much geospatial variation in locoregional therapy may be attributed to region vs patient factors.

**Design, Setting, and Participants:**

This retrospective cross-sectional study included women aged 70 years or older who were diagnosed with HR^+^/ERBB2-negative (ERBB2^−^) breast cancer from January 1, 2013, to December 31, 2017. Data came from Surveillance, Epidemiology, and End Results–Medicare. Hierarchical multivariable modeling was used to evaluate the variance in deescalated care attributable to 4 domains, ie, (1) random, (2) region (health service area [HSA]), (3) patient factors, and (4) unexplained. Patient factors included age, frailty (validated claims-based measure), Charlson Comorbidity Index (CCI), and socioeconomic status (Yost index). Analyses were performed from January to October 2023.

**Exposure:**

HSA.

**Main Outcomes and Measures:**

Deescalated care, defined as omission of axillary surgery, RT, or both. Standard therapy was defined as lumpectomy, axillary surgery, and RT or mastectomy with axillary surgery. Multivariable logistic regression was used to identify factors associated with deescalated care receipt.

**Results:**

Of 9173 patients (mean [SD] age, 76.5 [5.2] years), 2363 (25.8%) were aged 80 years or older, 705 (7.7%) had frailty, and 419 (4.6%) had a CCI of 2 or greater. While 4499 (49.1%) underwent standard therapy, 4674 (50.9%) underwent deescalated therapy (1193 [13.0%] of the population omitted axillary surgery and 4342 [55.5%] of patients undergoing lumpectomy omitted RT). Of the total variance, random variation explained 27.3%, region/HSA explained 35.3%, patient factors explained 2.8%, and 34.5% was unexplained. In adjusted models, frailty and increased age were associated with a higher likelihood of undergoing deescalated therapy (frailty: odds ratio [OR], 1.70; 95% CI, 1.43-2.02; age, by 1-year increment: OR, 1.10; 95% CI, 1.09-1.11), but CCI was not. Patients in rural areas compared with those in urban areas (OR, 0.82; 95% CI, 0.68-0.99) and Asian and Pacific Islander patients compared with non-Hispanic White patients (OR, 0.68; 95% CI, 0.54-0.85) had a lower likelihood of undergoing deescalated therapy.

**Conclusions and Relevance:**

In this retrospective cross-sectional study of women aged 70 years or older diagnosed with T1N0 HR^+^/ERBB2^−^ breast cancer, region/HSA contributed more to the variation in deescalated therapy use than patient factors. Unexplained variation may be attributed to unmeasured characteristics, such as multidisciplinary environment and patient preference. Decision support efforts to address overtreatment should target regions with low rates of evidence-based deescalation.

## Key Points

**Question:**

To what extent does health service area contribute to variation in deescalated care in older adults with early-stage hormone receptor–positive breast cancer?

**Findings:**

In this cohort study with 9173 patients, most variation (61.8%) was attributed to random or unexplained factors. Measured factors, such as health service area (35.3%) and patient factors (2.8%), explained a minority of the variation.

**Meaning:**

These findings suggest that efforts to appropriately deescalate treatment in this patient population should focus on regions with particularly high usage rates, with ongoing work to define and address the unmeasured factors, such as multidisciplinary environment, that contribute to possible overtreatment.

## Teaser

This cohort study evaluates whether regional differences are associated with the likelihood of older patients receiving deescalated care for early-stage breast cancer.

## Introduction

Recommendations from Choosing Wisely^1^ and the National Comprehensive Cancer Network^2^ support the omission of sentinel lymph node biopsy (SLNB) and radiation therapy (RT) in select older adults with early-stage hormone receptor–positive (HR^+^) breast cancer. These recommendations draw from randomized clinical trial (RCT) data, notably from the PRIME II trial,^3^ which included women aged 65 years or older, and the Cancer and Leukemia Group B (CALGB) 9343^4^ trials, which included women aged 70 years or older. Both trials found that there was no survival decrement with the omission of RT in this older population who were prescribed endocrine therapy (ET). CALGB 9343 also supported the omission of axillary surgery in this population, as long-term follow-up data published in 2013 showed axillary recurrence rates of 3% among women without axillary surgery and RT at a median 12 years of follow-up. Similarly, randomized trials published in 2006^5^ and in 2012,^6^ which included patients with all disease subtypes and who were as young as 60 years, showed no difference in overall survival and axillary recurrence rates of 6% or less in those who had axillary surgery omitted.

Still, significant usage of these treatments persists.^7^ Even as studies expanding the age criteria in the omission of SLNB or RT into younger populations^8,9^ or examining the possibility of RT or ET as an either/or option^10^ have been accruing and reporting, approximately 65% of women aged 70 years or older with early-stage HR^+^ breast cancer still undergo RT, and 80% to 85% undergo axillary surgery, including more than 50% of women who have frailty.^7^

Despite the long history of data supporting deescalation of locoregional therapy in the field of breast cancer, uptake of deescalation practices has demonstrated significant geographic variation, starting with variable rates of breast conservation vs mastectomy documented in the 1980s.^11^ Little is known, however, about geographic variation in omission of RT and/or axillary surgery in women aged 70 years or older with HR^+^/ERBB2-negative (ERBB2^−^) breast cancer. As quantifying regional variation could inform de-implementation efforts of RT and/or axillary surgery in this population, we aimed to determine how much geospatial variation in locoregional therapy is attributed to health service area (HSA) vs patient factors.

## Methods

### Data Source

The Surveillance, Epidemiology, and End Results (SEER) Program, funded by the National Cancer Institute, collects cancer incidence, prevalence, and survival data for participating US cancer registries, representing approximately 35% of the US population.^12^ This analysis used data from the 2020 SEER-Medicare linkage. As this is a limited dataset, the project was deemed exempt and the need for informed consent was waived by the Mass General Brigham institutional review board. This report follows the Strengthening the Reporting of Observational Studies in Epidemiology (STROBE) reporting guideline.

### Patients

Women aged 70 years or older diagnosed with T1N0 HR^+^/ERBB2^− ^breast cancer, who had continuous Medicare Parts A and B coverage from 1 year prior to diagnosis through to 1 year after diagnosis, without health maintenance organization (HMO) enrollment, and who received locoregional therapy were identified. Given RCT data preceding the publication of the long-term follow-up of CALGB 9343^5,6,13^ supporting omission of axillary surgery and RT, patients diagnosed from January 1, 2013, to December 31, 2017, were included. Patients who had a previous history of breast cancer, diagnosis of bilateral breast cancer, breast cancer identified by autopsy or death certificate, unknown surgery type, unknown receptor status, or unknown tumor stage as well as those who underwent neoadjuvant chemotherapy or had neoadjuvant RT were excluded (eFigure in Supplement 1). Patients who were treated in an HSA that had fewer than 10 eligible patients were also excluded to allow for sufficient variation.

### Outcome Measure

The outcome of interest was receipt of deescalated treatment, defined as the omission of either axillary surgery or RT or both (ie, lumpectomy alone, mastectomy alone, lumpectomy with axillary surgery, or lumpectomy with RT). Standard treatment included patients who underwent lumpectomy with axillary surgery and RT or mastectomy with axillary surgery. *International Classification of Diseases, Ninth Revision *(*ICD-9*) and *International Statistical Classification of Diseases and Related Health Problems, Tenth Revision *(*ICD-10*) procedures and *Common Procedural Terminology *(*CPT*) codes in addition to the SEER variables were used to determine treatment received in the year after diagnosis.

### Variables

The key factor of interest was frailty, defined by the claims-based frailty indicator (CFI) developed by Kim et al.^4^ The CFI incorporates 93 variables defined by *ICD* diagnosis codes, *CPT* codes, and Healthcare Common Procedure Coding System coded in the 12 months prior to diagnosis, resulting in a value ranging from 0 to 1. A binary variable was created to indicate frailty status, with CFI of 0.2 or greater indicating frailty, as previously described.^14,15^

SEER race and ethnicity variables were combined to categorize participants as African American or Black, Asian or Pacific Islander, Hispanic or Latinx White, non-Hispanic or Latinx White, and other or unknown. Other or unknown race and ethnicity includes patients coded as American Indian, Asian Indian, Chamorran, Chinese, Fiji Islander, Filipino, Guamanian, Hawaiian, Hmong Kampuchean, Japanese, Korean, Laotian, Melanesian, Micronesian, New Guinean, Pakistani, Polynesian, Samoan, Tahitian, Thai, Tongan, Vietnamese, other Asian not otherwise specified, other Pacific Islander not otherwise specified, other, or unknown. Patient-level characteristics included year of diagnosis; age; Charlson-Deyo Comorbidity Index (0, 1, ≥2)^6,7^; socioeconomic status quintile using Yost Index (group 1, the lowest, to group 5, the highest), which includes factors such as education, income, and education^8^; tumor grade (1, 2, 3); tumor category (T1a, T1b, T1c); and tumor histology (invasive ductal carcinoma, invasive lobular carcinoma, or other). All patients were node negative. County characteristics included the area of residence (urban or rural) using the classification scheme of the 2013 Rural-Urban Continuum Codes and SEER region (West, Northeast, Midwest, or South). Geospatial variation was analyzed at the level of the HSA. Originally defined by a cluster analysis of where residents aged 65 years and older obtained routine short-term hospital care,^16^ HSAs have been modified over the years to ensure that they were at least 250 miles square miles and that none of the HSAs cross state boundaries.^17^ There were 187 HSAs included in SEER regions in our study years.

### Statistical Analysis

We used χ^2^ tests to compare descriptive characteristics by treatment type (deescalated vs standard treatment). Hierarchical multivariable models were used to examine geospatial variation in locoregional treatment patterns across health service areas (HSAs)^10^ that included 10 or more eligible patients. The total variance across HSAs in deescalated treatment received was attributed proportionally to 4 component variances, ie, (1) random (regardless of patients’ characteristics and HSA), (2) patient factors (frailty, Charlson-Deyo Comorbidity Index [CCI], chronologic age, Yost index, race and ethnicity, tumor grade, tumor category, tumor histology, and year of diagnosis), (3) region (HSA), and (4) unexplained. Data analyses were performed from January to October 2023.

The simplest null model (M0) by binomial distribution did not include any fixed or random effects and showed the variance attributed to randomness. A logistic regression model (M1) was conducted to derive patient factor variance by including patient characteristics as a fixed effect. HSA was further added as a random effect to the M1 model to obtain region variance in the M2 model. We defined incremental variance over their previous model (ie, M1 − M0 and M2 − M1) as variance attributable to each component. To stabilize the variance estimates, we used simulated percentages from all 87 HSAs and 9173 patients to calculate the variance of the distribution. In the simulation, we generated the percentages 1000 times for each HSA and 1000 times for each patient. Sensitivity analyses excluded HSAs with fewer than 30 cases, and patterns remained robust. A random-effects logistic model, accounting for patient factors as a fixed effect and adding HSAs as a random intercept, was run to estimate the association between frailty and receipt of deescalated treatment.

Given the associations found in our multivariable model, we performed subanalyses to understand locoregional treatment by race and ethnicity and urbanity. Sensitivity analyses were run excluding patients undergoing mastectomy, as high rates of axillary surgery in these patients have been previously reported.^18,19^ As measures of frailty and multimorbidity may have significant overlap, sensitivity analyses were also run to ensure adjusted model results were robust.

Tests of significance were 2-sided, with α set at .05. All analyses were performed using SAS software version 9.4 (SAS Institute) and R version 4.3.1 (R Foundation for Statistical Computing).

## Results

Of 9173 patients (mean [SD] age, 76.5 [5.2] years), 2363 (25.8%) were aged 80 years or older, 705 (7.7%) had frailty, and 419 (4.6%) had a CCI of 2 or greater (Table 1). There were 453 (4.9%) African American or Black participants, 461 (5.0%) Asian or Pacific Islander participants, 390 (4.3%) Hispanic or Latinx White participants, and 7783 (84.8%) non-Hispanic or Latinx White participants. Most lived in urban or semiurban areas (8338 [90.9%]) vs rural areas (835 [9.1%]). While 4499 (49.1%) underwent standard therapy, 4674 (50.9%) underwent deescalated therapy. A greater proportion of patients who underwent deescalated care had frailty, were aged 80 years or older, lived in urban areas, had grade 1 tumors, and had T1a or T1b tumors (*P* < .001 for all comparisons). Over the study period, there was an increase in deescalated therapy (1186 of 2023 [58.6%] in 2017 vs 578 of 1504 [38.4%] in 2013; *P* < .001).

**Table 1.** Baseline Patient Characteristics

Characteristic	No. (%)			*P* value
	Total (N = 9173)	Standard therapy (n = 4499 [49.1%])	Deescalated therapy (n = 4674 [50.9%])	
Frailty				
Yes	705 (7.69)	257 (5.7)	448 (9.6)	<.001
Age, y				
Mean (SD)	76.5 (5.2)	75.3 (4.4)	77.6 (5.7)	<.001
70-74	3992 (43.5)	2308 (51.3)	1684 (36.0)	
75-79	2818 (30.7)	1438 (32.0)	1380 (29.5)	
80-84	1548 (16.9)	567 (12.6)	981 (21.0)	
≥85	815 (8.9)	186 (4.1)	629 (13.5)	
Charlson-Deyo Comorbidity Index				
0	7761 (84.6)	3833 (85.2)	3928 (84.0)	.32
1	993 (10.8)	471 (10.5)	522 (11.2)	
≥2	419 (4.6)	195 (4.3)	224 (4.8)	
Socioeconomic status (Yost score)				
Group 1 (lowest)	784 (8.6)	383 (8.5)	401 (8.6)	.30
Group 2	1122 (12.2)	583 (13.0)	539 (11.5)	
Group 3	1660 (18.1)	820 (18.2)	840 (18.0)	
Group 4	2285 (24.9)	1106 (24.6)	1179 (25.2)	
Group 5 (highest)	3322 (36.2)	1607 (35.7)	1715 (36.7)	
Race and ethnicity				
African American or Black	453 (4.9)	239 (5.3)	214 (4.6)	.08
Asian or Pacific Islander	461 (5.0)	248 (5.5)	213 (4.6)	
Hispanic or Latinx White	390 (4.3)	190 (4.2)	200 (4.3)	
Non-Hispanic or Latinx White	7783 (84.9)	3785 (84.1)	3998 (85.5)	
Other or unknown^a^	86 (0.9)	37 (0.8)	49 (1.1)	
Year of diagnosis				
2013	1504 (16.4)	926 (20.6)	578 (12.4)	<.001
2014	1777 (19.4)	1034 (23.0)	743 (15.9)	
2015	1805 (19.7)	813 (18.1)	992 (21.2)	
2016	2064 (22.5)	889 (19.8)	1175 (25.1)	
2017	2023 (22.1)	837 (18.6)	1186 (25.4)	
Urban or rural status				
Urban	8338 (90.9)	4036 (89.7)	4302 (92.0)	<.001
Rural	835 (9.1)	463 (10.3)	372 (8.0)	
SEER region				
West	4323 (47.1)	2002 (44.5)	2321 (49.7)	<.001
Northeast	2038 (22.2)	981 (21.8)	1057 (22.6)	
Midwest	965 (10.5)	557 (12.4)	408 (8.7)	
South	1847 (20.1)	959 (21.3)	888 (19.0)	
Tumor grade				
1	3834 (41.8)	1769 (39.3)	2065 (44.2)	<.001
2	4388 (47.8)	2208 (49.1)	2180 (46.6)	
3	749 (8.2)	422 (9.4)	327 (7.0)	
Unknown	202 (2.2)	100 (2.2)	102 (2.2)	
Tumor category				
T1a (0.1-0.5 cm)	1253 (13.7)	552 (12.3)	701 (15.0)	<.001
T1b (>0.5-1.0 cm)	3221 (35.1)	1521 (33.8)	1700 (36.4)	
T1c (>1.0-2.0 cm)	4457 (48.6)	2329 (51.8)	2128 (45.5)	
Others	242 (2.6)	97 (2.2)	145 (3.1)	
Histology				
IDC	7012 (76.4)	3442 (76.5)	3570 (76.4)	<.001
ILC	1497 (16.3)	773 (17.2)	724 (15.5)	
Other	664 (7.2)	284 (6.3)	380 (8.1)	
Locoregional therapy				
Lumpectomy alone	861 (9.4)	NA	861 (18.4)	NA
Lumpectomy with axillary surgery	3481 (38.0)	NA	3481 (74.5)	
Lumpectomy with RT	262 (2.9)	NA	262 (5.6)	
Mastectomy alone	70 (0.8)	NA	70 (1.5)	
Lumpectomy with axillary surgery and RT	3223 (35.1)	3223 (71.6)	NA	
Mastectomy with axillary surgery	1276 (13.9)	1276 (28.4)	NA	

Abbreviations: IDC, invasive ductal carcinoma; ILC, invasive lobular carcinoma; NA, not applicable; RT, radiation treatment; SEER, Surveillance, Epidemiology, and End Results.

^a^ Other or unknown race/ethnicity includes patients coded as American Indian, Asian Indian, Chamorran, Chinese, Fiji Islander, Filipino, Guamanian, Hawaiian, Hmong Kampuchean, Japanese, Korean, Laotian, Melanesian, Micronesian, New Guinean, Pakistani, Polynesian, Samoan, Tahitian, Thai, Tongan, Vietnamese, other Asian not otherwise specified, Pacific Islander not otherwise specified, other, or unknown.

Local therapy included mastectomy in 1346 patients (14.7%; 70 [0.8%] underwent mastectomy alone, and 1276 [13.9%] underwent mastectomy with axillary surgery). A greater proportion of Asian or Pacific Islander patients underwent a mastectomy compared with non-Hispanic White patients (eTable 1 in Supplement 1). Similarly, a greater proportion of women living in rural areas underwent a mastectomy compared with those living in urban areas (eTable 2 in Supplement 1).

Of the 7827 patients who underwent a lumpectomy, 262 (3.3%) omitted axillary surgery, 3481 (44.5%) omitted RT, and 861 (11.0%) omitted both. Rates of omission of RT, SLNB, or both in patients undergoing lumpectomy were similar between non-Hispanic White patients and Asian or Pacific Islander patients (eTable 1 in Supplement 1). More rural patients undergoing lumpectomy omitted SLNB or RT compared with urban patients undergoing lumpectomy (eTable 2 in Supplement 1).

**Figure 1.**
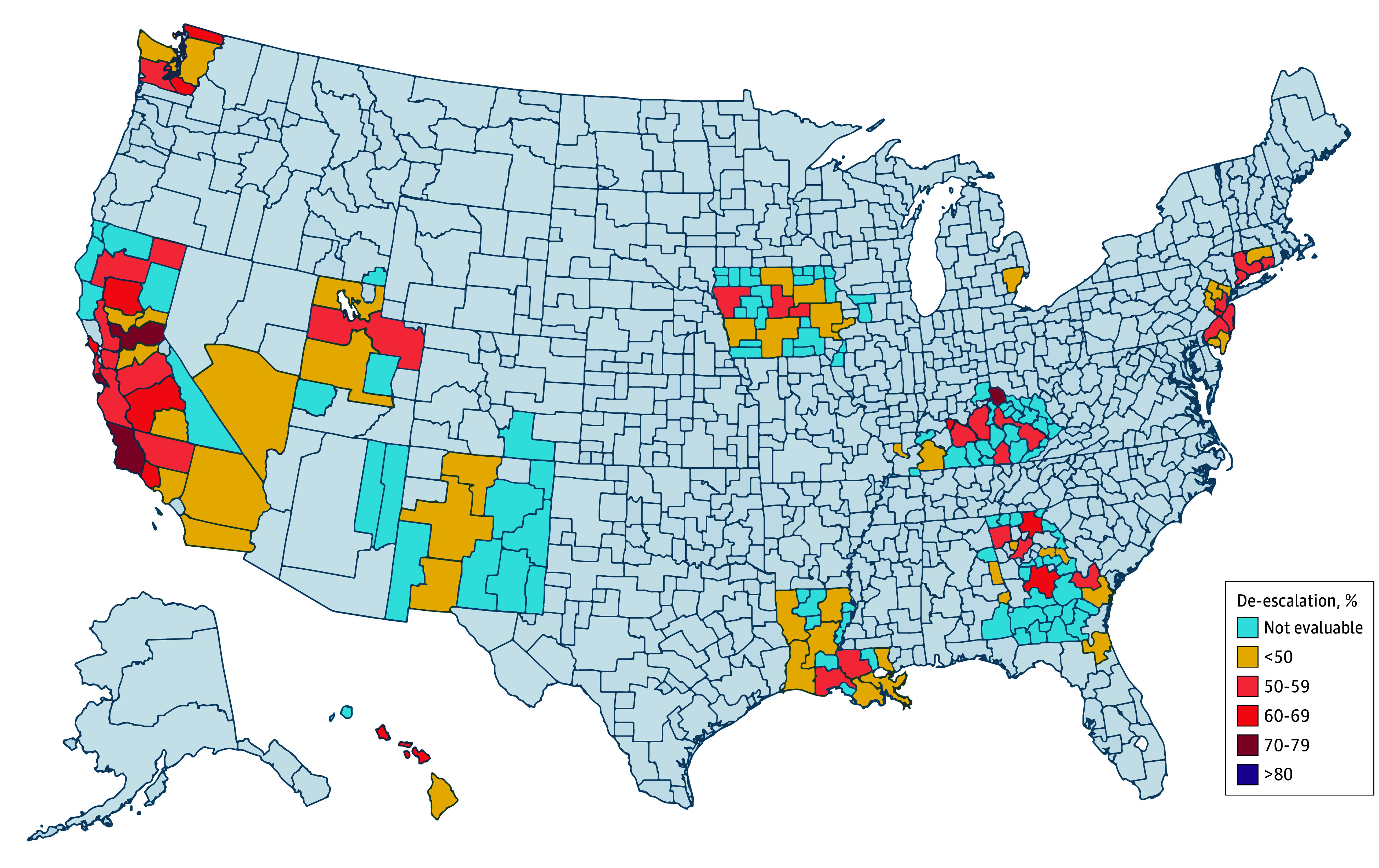
Chloropleth Map for Percentage of Women Aged 70 Years or Older With Hormone Receptor–Positive/ERBB2-Negative Breast Cancer Receiving Deescalated Locoregional Therapy in Surveillance, Evidence, and End Results Health Service Areas

**Figure 2.**
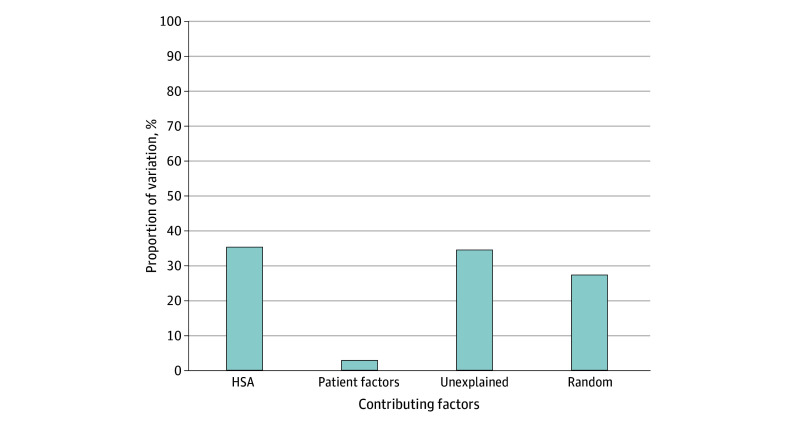
Proportion of Variance Attributable to Health Service Area (HSA) and Other Factors

Figure 1 shows the rates of deescalated treatment within each HSA. Across the 87 eligible HSAs, rates of deescalated therapy ranged from 23.3% to 79.7%. The HSAs with the highest and lowest deescalation rates can be found in Table 2. Sequential hierarchical models demonstrated that of the total variance, HSA explained 35.3%, while patient factors explained only 2.8% (Figure 2). Variation attributed to randomness and that which went unexplained by the measured factors in this dataset accounted for the majority (61.8%).

**Table 2.** Health Service Areas With the Highest and Lowest Rates of Deescalated Therapy

Health service area	Therapy rates, No. (%)	Total patients, No.
Highest rates of deescalated therapy		
Santa Barbara to San Luis Obispo, CA	145 (79.7)	182
Santa Cruz, CA	41(77.4)	53
Kenton (Covington) to Campbell, KY	31 (73.8)	42
Sacramento to Placer, CA	148 (71.2)	208
Honolulu to Maui, HI	77 (65.8)	117
Lowest rates of deescalated therapy		
St Tammany (Slidell) to Washington, LA	15 (37.5)	40
Sutter (Yuba City) to Yuba, CA	11 (34.4)	32
San Joaquin (Stockton) to Calaveras, CA	25 (33.8)	74
Polk (Des Moines) to Dallas, IA	31 (24.6)	126
Russell, AL	<11 (23.3)^a^	≤50
Highest rates of axillary surgery		
Bulloch to Emanuel, GA	18 (100.0)	18
Hawaii, HI	20 (100.0)	20
St Tammany (Slidell), to Washington, LA	24 (100.0)	24
Morgan to Greene, GA	37 (97.4)	38
Pierce, WA	108 (97.3)	111
Lowest rates of axillary surgery		
Bergen to Hudson (Jersey City), NJ	187 (74.8)	250
Fresno (Fresno) to Kings, CA	68 (66.0)	103
Kenton (Covington) to Campbell, KY	25 (65.8)	38
Fairfield, CT	91 (65.5)	139
Pulaski to Wayne, KY	11 (64.7)	17
Highest rates of radiation therapy^b^		
Cerro Gordo to Kossuth, IA	30 (76.9)	39
Polk (Des Moines) to Dallas, IA	72 (71.3)	101
Chatham (Savannah) to Liberty, GA	39 (67.2)	58
Sutter (Yuba City) to Yuba, CA	19 (65.5)	29
Utah (Provo) to Sanpete, UT	31 (64.6)	48
Lowest rates of radiation therapy^b^		
Sacramento (Sacramento) to Placer, CA	41 (22.0)	186
Hardin to Meade, KY	<11 (20.8)^a^	<25
Santa Cruz, CA	<11 (18.0)^a^	≤50
Ouachita (Monroe) to Morehouse, LA	<11 (15.8)^a^	<25
Santa Barbara (Santa Barbara) to San Luis Obispo, CA	27 (15.7)	172

^a^ Data coarsened to comply with Centers of Medicare & Medicaid Services cell size suppression policy.

^b^ Excluding patients undergoing mastectomy.

In adjusted models, frailty and increasing age were associated with a higher likelihood of undergoing deescalated therapy (frailty: odds ratio [OR], 1.70; 95% CI, 1.43-2.02; age, by 1-year increment: OR, 1.10; 95% CI, 1.09-1.11) (Table 3), but CCI was not. This finding was consistent in sensitivity analyses in models that included only frailty (ie, excluding CCI) and those including only CCI (excluding frailty). Higher tumor grade (grade 2 or 3 vs grade 1), T1c tumors (compared with T1b or T1a tumors), and later diagnosis year (2015-2017 vs 2013) were also significantly associated with an increased likelihood of undergoing deescalated therapy. Patients in rural areas, compared with those in urban areas, had a lower likelihood of undergoing deescalated therapy (OR, 0.82; 95% CI, 0.68-0.99), as did Asian and Pacific Islander patients compared with non-Hispanic White patients (OR, 0.68; 95% CI, 0.54-0.85). These associations were no longer significant in a sensitivity analysis that excluded patients undergoing mastectomy (eTable 3 in Supplement 1).

**Table 3.** Multivariable Logistic Regression Model for Receipt of Deescalated Care

Characteristic	OR (95% CI)	*P* value
Frail		
No	1 [Reference]	NA
Yes	1.70 (1.43-2.02)	<.001
Age (increase by 1 y)	1.10 (1.09-1.11)	<.001
Charlson-Deyo Comorbidity Index		
0	1 [Reference]	NA
1	1.11 (0.91-1.20)	.17
≥2	1.04 (0.84-1.29)	.74
Yost Index, quintile		
First (lowest SES)	1 [Reference]	NA
Second	0.82 (0.68-1.01)	.06
Third	0.92 (0.76-1.12	.28
Fourth	0.91 (0.75-1.09)	.30
Fifth (highest SES)	0.90 (0.75-1.09)	.29
Race and ethnicity		
African American or Black	0.83 (0.67-1.03)	.09
Asian or Pacific Islander	0.68 (0.54-0.85)	<.001
Hispanic or Latinx White	0.89 (0.71-1.12)	.29
Non-Hispanic or Latinx White	1 [Reference]	NA
Other or unknown^a^	1.21 (0.77-1.92)	.41
Urban or rural status		
Urban	1 [Reference]	NA
Rural	0.82 (0.68-0.99)	.04
Tumor grade		
1	1 [Reference]	NA
2	0.86 (0.78-0.97)	.002
3	0.73 (0.62-0.87)	<.001
Unknown	0.91 (0.67-1.24)	.57
Tumor category		
T1a	1 [Reference]	NA
T1b	0.87 (0.76-1.01)	.06
T1c	0.69 (0.60-0.79)	<.001
Unknown	0.91 (0.67-1.23)	.55
Histology		
IDC	1 [Reference]	NA
ILC	0.88 (0.78-0.99)	.04
Other or unknown	1.24 (1.05-1.48)	.01
Year of diagnosis		
2013	1 [Reference]	NA
2014	1.16 (0.99-1.34)	.05
2015	2.01 (1.74-2.33)	<.001
2016	2.28 (1.97-2.62)	<.001
2017	2.53 (2.19-2.93)	<.001

Abbreviations: IDC, invasive ductal carcinoma; ILC, invasive lobular carcinoma; NA, not applicable; OR, odds ratio; SES, socioeconomic status.

^a^ Other or unknown race/ethnicity includes patients coded as American Indian, Asian Indian, Chamorran, Chinese, Fiji Islander, Filipino, Guamanian, Hawaiian, Hmong Kampuchean, Japanese, Korean, Laotian, Melanesian, Micronesian, New Guinean, Pakistani, Polynesian, Samoan, Tahitian, Thai, Tongan, Vietnamese, other Asian not otherwise specified, Pacific Islander not otherwise specified, other, or unknown.

## Discussion

Our analysis found that the contribution of region/HSA to variation in locoregional treatment receipt in women with early-stage HR^+^/ERBB2^−^ disease was larger than patient-level factors, speaking to the higher-level forces that can affect practice. Certainly, the omission of specific treatments that lead to deescalated care differ by HSA. For instance, HSAs in Santa Barbara and Santa Cruz. California, had high rates of deescalated therapy, likely due to high rates of omission of RT in patients undergoing a lumpectomy. In contrast, an HSA in Kentucky was also in the top 5 for deescalated therapy, likely due to low axillary surgery rates. But the underlying care patterns can be influenced by a multitude of factors, and which ones underlie specific patterns within a given HSA remains unclear. Uneven distribution of health care services around the United States is well-known, showing areas in sore need of medical and radiation oncologists^20,21^ as well as surgeons.^22,23,24^ Cost variation in RT for breast cancer has also been documented, with the largest driver of cost variation consisting of location of care and individual clinicians,^25^ yet how access to and cost of care might affect de-implementation strategies remains largely unexplored. Older data would suggest that the operating surgeon is strongly associated with receipt of adjuvant RT,^26^ although more recent work examining the role of the individual surgeon in the omission of RT in the older adult population suggests that individual surgeons account for only a minority of practice variation (10%-13%).^27^ The malpractice environment is also highly varied by geography^28^ and may weigh into physician anxiety regarding recommendations of deescalated care.^29,30^

Unmeasured factors were associated with approximately 35% of the variation seen in deescalated therapy. Unmeasured variation can include variables from the disease level, such as margin status or presence of extensive ductal carcinoma in situ, through the patient level, such as maximizer or minimizer preferences toward health care,^31^ and up to the system level, such as treatment guidelines inherent to different hospital systems.^29^ Previous studies have demonstrated that physicians often think of axillary surgery and RT decisions as being interrelated and often factor in the likelihood of a patient adhering to ET.^29^ Locoregional treatment decisions are likely not attributable to one physician but may lie at the intersection of a team of surgery, radiation oncology, and medical oncology clinicians. HSAs were originally defined by the National Center for Health Statistics as an area of contiguous counties that are relatively self-contained with respect to hospital care.^32^ The nuances of multidisciplinary care may be attributable to regions as patients may go to different hospitals for treatment but may stay within a given region for the totality of care. Physician networks and working relationships may thus influence the intensity of therapy received. However, whether this is captured in our analysis in the variation at the level of the HSA or within the unmeasured variation (ie, variation that may be attributable to factors not captured in this analysis) is unclear. What is clear is that the highly complex nature of oncologic practice patterns illustrates the need for future work that examines practices at a level higher than individual practices or hospitals. Case studies at the level of the HSA may be productive, as the specific HSAs that had the highest and lowest rates of deescalated care in our study were not nested in a particular state (eg, California had HSAs with both high and low rates).

Patient factors in our analysis that were significantly associated with the receipt of deescalated locoregional therapy included frailty, chronologic age, rurality, and being an Asian or Pacific Islander individual. Pathologic factors, such as tumor grade, tumor category, histology, and year of diagnosis, were also significantly associated with the increased likelihood of undergoing deescalated therapy, consistent with multiple previous studies.^18,19,27,29,30,33^ The strong association between chronologic age and omission of SLNB and/or RT^7,18,19,34,35,36^ has also been previously demonstrated, as has the association between frailty and lower-intensity locoregional care,^7^ but as patient-level factors accounted for less than 3% of the variation, their overall contribution to treatment receipt was diminutive. Although comorbidities have been shown in previous studies as being significantly associated with lower rates of SLNB and RT,^35,37^ CCI was not significant in this analysis. While there is overlap in the comorbidities captured in the claims-based frailty indicators used and the CCI,^38^ in sensitivity analyses excluding frailty and changing CCI groupings, the association between CCI and deescalated care remained nonsignificant. Frailty, in models both with and without CCI, remained significantly associated with the outcome, demonstrating that the measures of frailty and multimorbidity used here distinguish between the constructs. As other studies have demonstrated, mastectomy is significantly associated with a higher likelihood of undergoing SLNB,^18,19^ possibly due to the fact that the CALGB 9343 trial^4^ and the trial by Martelli et al^6^ were done in patients undergoing breast conservation. In addition, surgeons may be anxious that final pathology on a mastectomy specimen may show a tumor significantly larger than on imaging, and thus they would lose their ability to do an SLNB, as this procedure relies on intact lymphatic drainage from the breast to the axilla. However, recommendations of omission of SLNB in older women with early-stage HR^+^/ERBB2^−^ disease do not exclude patients undergoing mastectomy^1,2,39^ as it is reasonable to omit SLNB and RT, as CALGB 9343 demonstrated.^4^

The lower odds of Asian and Pacific Islander patients receiving deescalated care were driven by higher rates of mastectomy with axillary surgery, as these odds lost significance in sensitivity analyses excluding patients undergoing mastectomy. In older analyses, an increasing trend in omission of RT in Asian and Pacific Islander patients undergoing lumpectomy was noted.^40^ Higher rates of mastectomy have also been previously noted in the Asian and Pacific Islander population compared with non-Hispanic White women,^41,42,43^ possibly driven by factors such as the belief that it is safer^44^ and simpler^45^; in addition, eligibility for breast conservation is contingent on breast-to-tumor ratio, and smaller breast size in some Asian and Pacific Islander women may be a factor.

Perhaps counterintuitively, patients in rural areas also had lower odds of receiving deescalated care. Previous literature has shown that distance from radiation facility^46,47^ and rurality^48,49,50^ are significantly associated with a lower likelihood of receipt of adjuvant RT. However, similar to past studies,^43,51^ mastectomy rates were noted to be higher in rural patients compared with urban patients. As noted in the Asian and Pacific Islander population, the lower likelihood of omission of axillary surgery in patients undergoing mastectomy ^18,19^ appeared to be driving the lower likelihood of deescalated care, thus illustrating the interrelated nature of choices of breast surgery, axillary surgery, and RT. As the distance needed to travel for RT can represent a significant barrier to breast conservation, mastectomy rates may be higher because physicians and patients believe that mastectomy is the only alternative to lumpectomy plus RT. Safely deescalating therapy in populations with high mastectomy rates may thus include efforts to (1) decrease the persistently high SLNB rates in older women with early-stage, HR^+^/ERBB2^−^ cancers undergoing mastectomy and (2) increase breast conservation rates by educating physicians and patients that lumpectomy without RT is a safe option.

### Limitations

This study has limitations, including those inherent to the use of the SEER-Medicare dataset, which includes reliance on coding in SEER-Medicare data, time lapse from diagnosis to release of the dataset, and restriction to SEER areas, with some regions not well represented. Our modeling also restricted our analysis to HSAs with a sufficient number of eligible patients in the study timeframe, and HSAs treating very low volumes of older patients with early-stage HR^+^/ERBB2^−^ disease may display variation in deescalation rates at even greater extremes that what was found here. In addition, we chose to focus on HSA-level variation rather than on surgeon-level variation within the HSA, as the number of HSAs with sufficient variance at the surgeon level were limited. As Medicare Part D was not part of our dataset, we were unable to include ET receipt in our analysis. Furthermore, we were unable to account for clinician bias and patient preferences given the nature of this dataset.

## Conclusions

In this retrospective cross-sectional study, more practice pattern variation was attributed to region than to patient-level factors in this population that is at risk for overtreatment, but there remained a substantial proportion of unexplained variation. Further qualitative and case-study approaches are needed to identify factors above the level of the patient and physician that have facilitated high levels of deescalated care and, in turn, to guide quality improvement and decision support efforts in regions with low rates of evidence-based deescalation.

## Notes

Supplement 1. **eFigure.** Flow Diagram **eTable 1. **Locoregional Therapy Breakdown for Asian and Pacific Islander Women **eTable 2.** Locoregional Therapy Breakdown by Urbanity **eTable 3.** Multivariable Logistic Regression Model for Receipt of Deescalated Care (Excluding Patients Undergoing Mastectomy)

Supplement 2.
Data Sharing Statement
