# Supplementary material for: Regional Variation in Deescalated Therapy in Older Adults With Early-Stage Breast Cancer
Source: JAMA Netw Open. 2024 Oct 24;7(10):e2441152. doi: 10.1001/jamanetworkopen.2024.41152 (PMC11581488; doi:10.1001/jamanetworkopen.2024.41152)
Supplement: Supplement 1. — eFigure. Flow Diagram eTable 1. Locoregional Therapy Breakdown for Asian and Pacific Islander Women eTable 2. Locoregional Therapy Breakdown by Urbanity eTable 3. Multivariable Logistic Regression Model for Receipt of Deescalated Care (Excluding Patients Undergoing Mastectomy) [file jamanetwopen-e2441152-s001.pdf]

Supplemental Online Content

Minami CA, Dey T, Chen Y, et al. Regional variation in deescalated therapy in older adults with early-stage breast cancer. *JAMA Netw Open.* 2024;7(10):e2441152. doi:10.1001/jamanetworkopen.2024.41152

eFigure. Flow Diagram

eTable 1. Locoregional Therapy Breakdown for Asian and Pacific Islander Women

eTable 2. Locoregional Therapy Breakdown by Urbanity

eTable 3. Multivariable Logistic Regression Model for Receipt of Deescalated Care (Excluding Patients Undergoing Mastectomy)

This supplemental material has been provided by the authors to give readers additional information about their work.

© 2024 Minami CA et al. *JAMA Network Open.*

eFigure. Flow Diagram

Initial total (Primary Site: Breast) = 528,117

↓

Exclude those with histologies not consistent with epithelial origin or lobular carcinoma in situ = 495,979

↓

Year 2013 ~ 2017 =280,582

↓

Exclude male patients = 277,908

↓

Exclude patients <70 years of age = 141,684

↓

Exclude patients with metastatic disease = 138,485

↓

Exclude patients with breast cancer diagnosis prior to index diagnosis = 97,148

↓

Exclude patients with breast cancer diagnosis diagnosed by autopsy or death certificate = 95,952

↓

Exclude patients with bilateral breast cancer = 95,936

↓

Exclude patients with radiation given before surgery = 95,788

↓

Exclude patients with unknown surgery type = 60,883

↓

Patients with Stage I, HR+ first primary breast cancer; node negative or unknown; HR+ (ER+ or PR+) AND HER-2 status is negative or unknown = 23,712

↓

Exclude patients who lacked Medicare part A and B coverage for the 1 year prior to diagnosis and year following diagnosis; patients with lack of continuous Medicare part A and B coverage; patients with HMO enrollment during study period; patients who underwent neoadjuvant chemotherapy within 6 months = 12,325

↓

Exclude patients with neither standard nor de-escalated therapy = 10,601

↓

Exclude patients treated in low-volume health service areas (HSAs) (<11 cases) = 9,173

© 2024 Minami CA et al. *JAMA Network Open.*

eTable 1. Locoregional Therapy Breakdown for Asian and Pacific Islander Women

	Non-Hispanic Whites	Asian/Pacific Islanders
**Locoregional Therapy**	**N=7,783 (84.8%)**	**N=461 (5.0%)**
Lumpectomy alone	720 (9.3%)	42 (9.1%)
Lumpectomy + axillary surgery	2,999 (38.5%)	148 (32.1%)
Lumpectomy + RT	226 (2.9%)	15 (3.3%)
Mastectomy Alone	53 (0.68%)	<11 (<2.0%)
Lumpectomy + axillary surgery + RT	2,758 (35.4%)	139 (30.2%)
Mastectomy + axillary surgery	1,027 (13.2%)	109 (23.6%)

*data coarsened to comply with CMS cell size suppression policy

© 2024 Minami CA et al. *JAMA Network Open.*

eTable 2. Locoregional Therapy Breakdown by Urbanity

	**Urban**	**Rural**
**Locoregional Therapy**	**N=8,338 (90.9%)**	**N=835, (9.1%)**
Lumpectomy alone	807 (9.7%)	>50 (>6.0%)*
Lumpectomy + axillary surgery	3,176 (38.1%)	305 (36.5%)
Lumpectomy + RT	251 (3.0%)	11 (1.3%)
Mastectomy Alone	68 (0.8%)	<11(<0.2)*
Lumpectomy + axillary surgery + RT	2,936 (35.2%)	287 (34.4%)
Mastectomy + axillary surgery	1,100 (13.2%)	176 (21.1%)

*data coarsened to comply with CMS cell size suppression policy

Abbreviations: **RT,** radiation therapy

© 2024 Minami CA et al. *JAMA Network Open.*

eTable 3. Multivariable Logistic Regression Model for Receipt of Deescalated Care (Excluding Patients Undergoing Mastectomy)

Characteristic	OR (95% CI)	p-value
**Frail**		
No	REF	
Yes	1.82 (1.5 – 2.23)	**<0.001**
**AGE** (increase by 1 year)	1.12 (1.11 – 1.13)	
**Charlson Comorbidity Index**		
0	REF	
1	1.28 (1.08 – 1.50)	**0.004**
>2	1.16 (0.90 – 1.48)	0.253
**Yost Index**		
Quintile 1 (lowest SES)	REF	
Quintile 2	0.81 (0.64 – 1.03)	0.08
Quintile 3	0.84 (0.68 – 1.05)	0.13
Quintile 4	**0.78 (0.63 – 0.98)**	**0.03**
Quintile 5 (highest SES)	**0.74 (0.60 – 0.92)**	**0.008**
**Race/Ethnicity**		
Asian or Pacific Islander	0.86 (0.67 – 1.12)	0.27
Black	0.81 (0.64 – 1.04)	0.10
Hispanic White	0.81 (0.63 – 1.03)	0.09
Non-Hispanic White	REF	
Other/Unknown	1.224(0.72 – 2.08)	0.46
**Urban/Rural Status**		
Urban	REF	
Rural	0.92 (0.74 – 1.14)	0.43
**Tumor Grade**		
1	REF	
2	**0.87 (0.78 – 0.96)**	**0.008**
3	**0.74 (0.61 – 0.90)**	**0.002**
Unknown	0.98 (0.69 – 1.4)	0.91
**Tumor Category**		
T1a	REF	
T1b	**0.75 (0.64 – 0.88)**	**<0.001**
T1c	**0.65 (0.56 – 0.76)**	**<0.001**
Unknown	1.15 (0.80 – 1.67)	0.45
**Histology**		
IDC	REF	
ILC	0.97 (0.84 – 1.11)	0.606
Other/Unknown	1.29 (1.06 – 1.56)	**0.012**

© 2024 Minami CA et al. *JAMA Network Open.*

Characteristic	OR (95% CI)	p-value
**Year of Diagnosis**		
2013	REF	
2014	1.13 (0.96 – 1.32)	0.15
2015	**2.09 (1.78 – 2.46)**	**<0.001**
2016	**2.44 (2.07 – 2.86)**	**<0.001**
2017	**2.69 (2.29 – 3.16)**	**<0.001**

Abbreviations: SES = socioeconomic status, OR = Odds Ratio, CI = Confidence Interval, REF = reference**;** Other/Unknown ethnicity: patients coded as American Indian, Chinese, Japanese, Filipino, Hawaiian, Korean, Vietnamese, Laotian, Hmong Kampuchean, Thai, Asian Indian, Pakistani, Micronesian, Chamorran, Guamanian, Polynesian, Tahitian, Samoan, Tongan, Melanesian, Fiji Islander, New Guinean, Other Asian Not Otherwise Specified (NOS), Pacific Islander NOS, Other, or Unknown in SEER; IDC = invasive ductal carcinoma, ILC = invasive lobular carcinoma

© 2024 Minami CA et al. *JAMA Network Open.*
