# Supplementary material for: Regional Variation in Deescalated Therapy in Older Adults With Early-Stage Breast Cancer
Source: JAMA Netw Open. 2024 Oct 24;7(10):e2441152. doi: 10.1001/jamanetworkopen.2024.41152 (PMC11581488; doi:10.1001/jamanetworkopen.2024.41152)
Supplement: Supplement 2. — Data Sharing Statement [file jamanetwopen-e2441152-s002.pdf]

Data Sharing Statement

Minami. Regional Variation in Deescalated Therapy in Older Adults With Early-Stage Breast Cancer. *JAMA Netw Open*. Published October 24, 2024.
doi:10.1001/jamanetworkopen.2024.41152

Data
Data available: No

Additional Information
Explanation for why data not available: The SEER-Medicare database is owned and managed by the National Cancer Institute. Information on how to obtain these data is available here: https://healthcaredelivery.cancer.gov/seermedicare/obtain/
